# Baseline immunity and impact of chemotherapy on immune microenvironment in cervical cancer

**DOI:** 10.1038/s41416-020-01123-w

**Published:** 2020-10-22

**Authors:** Yi Zhang, Minhua Yu, Ying Jing, Jiejun Cheng, Caiyan Zhang, Lin Cheng, Haijiao Lu, Mei-Chun Cai, Jie Wu, Wenjing Wang, Weihua Lou, Lihua Qiu, Li Tan, Huaiwu Lu, Xia Yin, Guanglei Zhuang, Wen Di

**Affiliations:** 1 State Key Laboratory of Oncogenes and Related Genes, Department of Obstetrics and Gynecology, Ren Ji Hospital, School of Medicine, Shanghai Jiao Tong University, Shanghai, China; 2 Shanghai Key Laboratory of Gynecologic Oncology, Ren Ji Hospital, School of Medicine, Shanghai Jiao Tong University, Shanghai, China; 3 Department of Radiology, Ren Ji Hospital, School of Medicine, Shanghai Jiao Tong University, Shanghai, China; 4 State Key Laboratory of Oncogenes and Related Genes, Shanghai Cancer Institute, Ren Ji Hospital, School of Medicine, Shanghai Jiao Tong University, Shanghai, China; 5 Department of Pathology, The Affiliated Hospital of Qingdao University, Qingdao, China; 6 Interdisciplinary Research Center on Biology and Chemistry, Shanghai Institute of Organic Chemistry, Chinese Academy of Sciences, Shanghai, China; 7 Department of Gynecologic Oncology, Sun Yat-Sen Memorial Hospital, Sun Yat-Sen University, Guangzhou, China

**Keywords:** Cervical cancer, Tumour immunology

## Abstract

**Background:**

We aimed to comprehensively evaluate the immunologic landscape at baseline and upon chemotherapy in cervical cancer. The information should aid ongoing clinical investigations of checkpoint blockade immunotherapies in this disease setting.

**Methods:**

A series of 109 cervical carcinoma patients was retrospectively assayed before and after neoadjuvant chemotherapy. Tumour-infiltrating immune markers (CD3, CD4, CD8, CD20, CD56, CD68, PD-1, PD-L1) were assessed by immunohistochemistry. RNA sequencing analysis was performed on matched pre- and post-treatment fresh-frozen tissues.

**Results:**

At diagnosis, diverse immune cell types including CD20+ B cells, CD3+ T cells, CD56+ natural killer (NK) cells, and CD68+ macrophages were detected in different proportions of cervical carcinoma. Unsupervised hierarchical clustering evidently showed that CD4+ and CD8+ T cell abundance correlated with PD-L1 expression. Based on the immune infiltration patterns, the patients could be stratified into four groups with prognostic relevance, namely, ‘immuno-active’, ‘immuno-medial’, ‘immuno-NK’, and ‘immuno-deficient’. Neoadjuvant chemotherapy was associated with increased CD4, CD8, CD20, and CD56 signals, most prominently in good responders. Transcriptomic data corroborated the improved anticancer immunity and identified immunosuppressive CD200 upregulation following chemotherapeutic intervention.

**Conclusions:**

A subset of cervical cancer harbours active immune microenvironment, and chemotherapy treatment may further exert locoregional immunostimulation. Immune checkpoint inhibitors as combination or maintenance therapies warrant future exploration in clinic.

## Background

Cervical cancer is a significant cause of women’s mortality with approximately 569,847 new cases and 311,365 deaths annually worldwide.^1^ Currently, patients with advanced cervical cancer have limited therapeutic options. In recent years, extensive epidemiological, laboratory, and clinical investigations have been undertaken to tackle this life-threatening problem. One notable progression is the ground-breaking discovery of high-risk human papillomavirus (HPV) as a major aetiological factor for cervical cancer.^2^ The subsequent prophylactic HPV vaccination and effective screening of precancerous lesions followed by preventive treatment have yielded a dramatic reduction in the late-stage disease incidence.^3,4^ Although we envision that cervical cancer will be eventually eliminated with these efforts, until it can be optimistically accomplished after decades,^5^ basic scientific advances still need to be made and novel lifesaving medicines are imminently desired to overcome this global threat of public health.^6,7^

It has been well established that tumour microenvironment, especially the immune milieu, plays a crucial role in modulating disease progression and response to anticancer therapies.^8–10^ As expected, cellular and molecular indicators of positive immune activities are typically associated with long-term patient survival, and vice versa.^11,12^ Indeed, numerous studies have identified certain immune contexture or immunity-related gene signatures as prognostic biomarkers in a wide spectrum of human malignancies.^13–16^ Along similar lines, accumulating data suggest that the baseline immunologic state within tumour lesions determines the clinical outcome following pharmacological interventions, ranging from conventional chemotherapeutics to targeted compounds.^17–19^ These anticancer agents may in turn trigger immunogenic cell death and alter the composition and phenotype of intratumoural immune infiltrates.^20–22^ By exerting cytotoxic effects, many anti-neoplastic drugs often have the tendency to stimulate the innate and acquired immune system, thereby facilitating tumour eradication.^23–25^ In fact, the ultimate therapeutic efficacy as a result of administered regimens sometimes hinges on their capacity to engage functional immune circuitries and restore immunosurveillance.^26–29^ Therefore, malignant cells commonly co-opt multiple evasion mechanisms to avoid immune attack,^30–33^ and a rational approach unleashing the immunoreactivity holds considerable promise for potentially curative remedies, as exemplified by the recent emergence of cancer immunotherapies to reinstate the immunological control of diverse neoplasms.^34–36^

Hallmarked by HPV-driven carcinogenesis, cervical cancer is presumed to possess immunogenicity by nature.^37–39^ Meanwhile, persistent viral infection could also induce host immune tolerance, thus leading to a more complicated scenario.^40,41^ Surprisingly, in-depth characterisation of its immunologic landscape has been rather scanty with only a few reports focussing on specific cell subsets.^42–45^ We reasoned that a more thorough understanding of the immune components and their interrelation with empirical treatments would provide enormous opportunities for improving patient management and optimising therapeutic protocols. In this study, using immunohistochemical (IHC) staining and RNA sequencing (RNA-seq), we systematically surveyed various immune cell populations present in different stages of cervical cancer at baseline or upon neoadjuvant chemotherapy (NACT). These integrated analyses allowed for a critical evaluation of tumour-infiltrating immune profiles and might contribute to the ongoing development of immunomodulatory therapies in cervical cancer.

## Methods

### Patient cohort

The study was conducted in accordance with ethical guidelines of the U.S. Common Rule and was approved by the Ethics Committee of Ren Ji Hospital. Appropriate written informed consent was obtained from each patient. All patients were treated at the Department of Obstetrics and Gynecology, Ren Ji Hospital, and their clinical records and tissue specimens were retrospectively retrieved. Formalin-fixed and paraffin-embedded (FFPE) sections were obtained in pathologic examination. For RNA-seq analysis, fresh-frozen tumour tissues were collected during diagnostic biopsy (pre-chemotherapy) and debulking surgery (post-chemotherapy). A total of 14 patients (28 paired samples) were assayed. Magnetic resonance imaging (MRI) data were provided by the Department of Radiology, Ren Ji Hospital. Detailed clinical characteristics of the patient cohort are described in Supplementary Table 1.

### Chemotherapy response evaluation

The clinical response to NACT was assessed according to the Response Evaluation Criteria in Solid Tumours. The evaluation was performed by an experienced radiologist (J.C.) on the basis of MRI images following 1–2 cycles of chemotherapy treatment. A complete response (CR) was defined as the disappearance of the initial lesions. A partial response (PR) was defined as the detection of at least a 30% reduction in the sum of the longest dimensions of the primary tumours. Progressive disease (PD) was defined as a >20% increase in the sum of the longest dimensions of the target lesions or the development of new lesions. Stable disease (SD) implied that none of the above applied. Patients with CR or PR were defined as good responders, and patients with SD or PD were defined as poor responders.

### Immunohistochemistry

IHC was performed on 5-μm-thick FFPE tissue sections. Slides were baked, deparaffinised in xylene, passed through graded alcohols, and antigen retrieved with 10 mM citrate buffer, pH 6.0 in a steam pressure cooker. Pre-processed tissues were treated with peroxidase block (Dako) to quench endogenous peroxidase activity, blocked using protein block (Dako), and incubated with primary antibodies (Supplementary Table 2). Slides were then washed in 50 mM Tris-HCl, pH 7.4 and incubated with horseradish peroxidase-conjugated secondary antibodies. Immunoperoxidase staining was developed using the DAB system according to the manufacturer’s instructions (Dako). Slides were counterstained with haematoxylin, dehydrated in graded alcohol and xylene, and cover-slipped using mounting solution.

### IHC staining quantification

Areas of necrosis or artefacts were ignored. Microscopically, the cell membrane in the slices was stained. The slides were examined using a bright field microscope and were scored using a four-point scale. First, for progression-free survival (PFS) survival analysis, the immune cellular staining of each antibody was semi-quantitatively scored as ‘−’ (no or <5% positive cells), ‘+’ (5–25% positive cells), ‘++’ (26–50% positive cells), and ‘+++’ (>50% positive cells). Both tumour and immune cell staining of programmed death-ligand 1 (PD-L1) were scored. The IHC signals were enumerated in ten random ×20 fields, and cell counts were normalised to the area of tumour tissues. The samples with staining scores of ‘−’ were considered as the negative group, whereas those with staining scores of ‘+’, ‘++’, and ‘+++’ were combined into the positive group. Second, in order to perform correlation analysis and quantitative comparison before and after NACT accurately, the slides were also scanned with an Aperio ScanScope system (Leica Biosystems) and quantified using the Aperio ImageScope software v12.1 with Positive Pixel Count v9 (PPCv9) algorithm for statistical analysis.

### RNA-seq and analysis

We performed RNA-seq analysis on 14 patients (28 samples) with matched pre- and post-chemotherapy fresh-frozen tissues. Total RNA was extracted from shavings of fresh-frozen specimens using the RNeasy Plus Kit (Qiagen) according to the manufacturer’s protocol. RNA purity and integrity were assessed by the NanoPhotometer spectrophotometer (Implen) and RNA Nano 6000 Assay Kit on Bioanalyzer 2100 system (Agilent Technologies), respectively. Total RNAs with RNA Integrity Number of >8 were subjected to next-generation sequencing. Total amount of 3 µg RNA for each sample was used as input materials for library preparation with the NEBNext Ultra Directional RNA Library Prep Kit (NEB). The index-coded libraries were clustered on a cBot Cluster Generation System using the TreSeq PE Cluster Kit v3-cBot-HS (Illumina) and sequenced on an Illumina Hiseq X Ten platform to generate 125 bp paired-end reads (Novogene). Clean data were obtained from FastQ raw data by removing adapter, poly-N sequences, and low-quality reads. All the downstream analyses were based on the clean data with high quality. Index of the reference genome was built using Bowtie v2.0.6 and paired-end clean reads were aligned to the reference genome (Ensembl hg38 human genome) using TopHat v2.0.9.^46^ The mapped reads of each sample were assembled by Cufflinks (v2.1.1) in a reference-based approach.^47^ Differential expression analysis was performed using Cuffdiff (v2.1.1). P-values were adjusted using the Benjamini–Hochberg procedure for controlling the false discovery rate. Genes with an adjusted *P* value of <0.05 were considered differentially expressed. The sequencing data have been deposited in the NCBI BioProject database (http://www.ncbi.nlm.nih.gov/bioproject/) under the accession number SRP173984.

### Statistical analysis

Statistical analyses were performed with the R language and Graphpad Prism 6. Unsupervised hierarchical clustering was conducted to define the immune subtypes based on the evaluated markers. Pearson correlation analysis was used to test the associations between different immune measurements. Cumulative survival rate was calculated by the Kaplan–Meier method and analysed by log-rank test. Cox proportional models were used to determine the hazard ratio that represents the relative risk of events among patients in the different groups. Gene ontology and pathway analyses were performed using Metascape (http://metascape.org).^48^ Single sample gene set enrichment analysis implemented in the Bioconductor ‘GSVA’ package was applied to generate compound scores for the indicated gene signatures.^49^ CIBERSORT was employed to estimate the relative abundance of diverse immune cell infiltrates from gene expression profiles.^50^ Antigen receptor repertoire present in bulk RNA-seq data was inferred by MiXCR.^51^ Comparisons between two conditions were based on two-sided Student’s *t* test. *P* values of <0.05 were judged to be statistically significant.

## Results

### Patient characteristics

The study cohort contained 109 cases of cervical cancer with high-quality FFPE tissues and clinicopathological information available (Supplementary Table 1). The median age of the patients was 52 years (range, 25–83 years). The histological diagnosis was mainly squamous cell carcinoma (89.9%) and adenocarcinoma (9.2%) of different International Federation of Gynecology and Obstetrics stages (IA–IVA). Forty (36.7%) and sixty-nine (63.3%) subjects received upfront radical hysterectomy and NACT followed by surgery or chemoradiotherapy, respectively (Supplementary Fig. 1a). We were able to obtain 92 treatment-naive samples and 60 chemo-exposed specimens from the diagnostic biopsies or surgical procedures, among which 43 pairs were matched pre- and post-NACT tissues. In total, 152 (92 treatment-naive and 60 chemo-exposed) FFPE blocks underwent IHC examination, and 28 fresh-frozen tumours (14 pre-NACT and 14 post-NACT) from the NACT group were subjected to RNA-seq analysis (RJCC1–14, all squamous cell carcinomas).

### Patterns of baseline immune infiltrates in cervical cancer

**Fig. 1:**
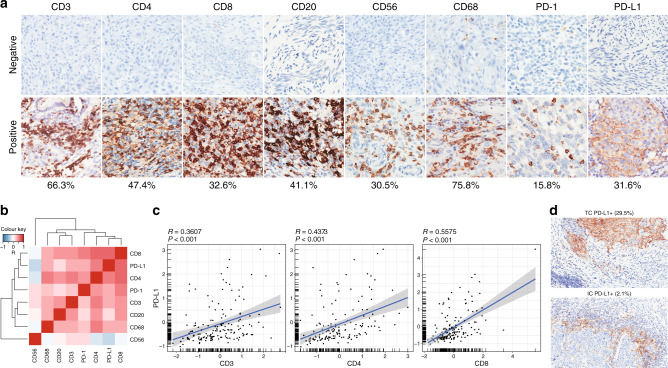
Patterns of baseline immune infiltrates in cervical cancer. **a** Representative images (×20) of negative or positive immunohistochemical staining with antibodies against CD3, CD4, CD8, CD20, CD56, CD68, PD-1, and PD-L1. The percentage of cervical cancer samples with positive immunostaining at baseline was shown. **b** Correlation matrix heatmap followed by unsupervised hierarchical clustering of evaluated immune markers in 92 untreated tumours. *R* indicates Pearson correlation coefficient. **c** Scatterplots with linear regression line and shaded 95% confidence region between PD-L1 staining intensity and CD3, CD4, or CD8 staining intensity. *R* indicates Pearson correlation coefficient. **d** The percentage of cervical cancer samples showing positive PD-L1 staining in tumour cells (TC) or immune cells (IC).

To systematically analyse the immune makeup of cervical cancer, CIBERSORT,^50^ a computational method for inferring the relative abundance of diverse cell infiltrates from bulk tumour transcriptomes, was initially conducted on the gene expression data (RJCC1–14, 28 data sets) profiled by RNA-seq. This framework pinpointed that the major representative immune cell types were B cells, T cells, natural killer (NK) cells, and macrophages (Supplementary Fig. 1b). Based on these findings, we assembled a panel of monoclonal antibodies to probe each subset-specific marker, as well as immune checkpoint molecules including programmed death-1 (PD-1) and PD-L1 (Supplementary Table 2). These eight immunologic parameters displayed divergent positive staining ratios in the 92 untreated samples. We observed CD3+ pan T cells (66.3%), CD4+ helper T cells (47.4%), CD20+ B cells (41.1%), and CD68+ macrophages (75.8%) in a prevalent population of cervical tumours, whereas CD8+ cytotoxic T cells (32.6%), CD56+ NK cells (30.5%), PD-1 (15.8%), and PD-L1 (31.6%) signals were restricted to a smaller fraction of cancer patients (Fig. 1a). While most immune markers were comparable between cervical adenocarcinoma and squamous cell carcinoma, histotype-specific CD20 and CD56 positivity was noted (Supplementary Fig. 2).

In order to better understand the complex immune characteristics in cervical cancer, we quantified the immune stains and assessed their interrelationships by analysing pairwise correlation between the evaluated variables. Unsupervised hierarchical clustering of Pearson correlation coefficients (*R*) was visualised in a heatmap, which identified a dominant array of co-modulated markers, including CD3, CD4, CD8, CD20, CD68, PD-1, and PD-L1 (Fig. 1b). There was a statistically significant positive correlation between PD-L1 intensity and CD3+, CD4+, or CD8+ tumour-infiltrating lymphocytes (TILs) (Fig. 1c), consistent with the known role of T cell-derived cytotoxicity as a driver of PD-L1 expression.^52^ Among the 29 cases (31.6%) showing PD-L1 staining, PD-L1 was mostly expressed on tumour cell surface (29.5%) and only sporadically detected in immune cells (2.1%; Fig. 1d). Taken together, these results indicated that cervical cancer at baseline contained both innate and adaptive immune cells, as well as immune checkpoint expression within the tumour microenvironment.

### Association of immune infiltrates with patient prognosis

**Fig. 2:**
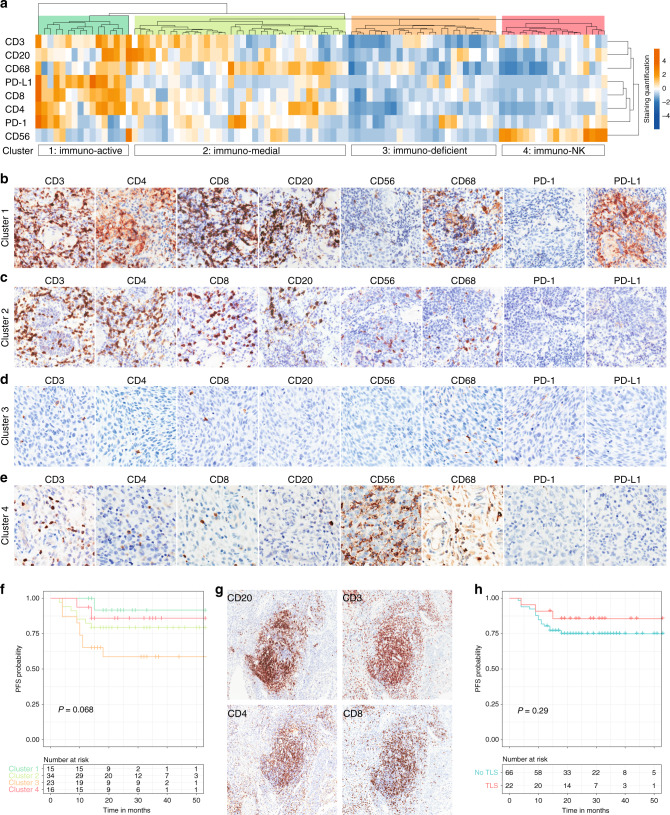
Association of immune infiltrates with patient prognosis. **a** Unsupervised hierarchical clustering of 92 treatment-naive tumour samples based on the quantification of evaluated immune markers. Four clusters were defined, which were named ‘immuno-active’ (cluster 1), ‘immuno-medial’ (cluster 2), ‘immuno-deficient’ (cluster 3), and ‘immuno-NK (cluster 4). **b** Representative images of immunohistochemical staining for the ‘immuno-active’ cluster. **c** Representative images of immunohistochemical staining for the ‘immuno-medial’ cluster. **d** Representative images of immunohistochemical staining for the ‘immuno-deficient’ cluster. **e** Representative images of immunohistochemical staining for the ‘immuno-NK cluster. **f** Prognostic value of immune subtyping in cervical cancer as indicated by Kaplan–Meier curves of progression-free survival (PFS). *P* value was based on the log-rank test. **g** Immunohistochemical staining of the indicated immune markers in typical tertiary lymphoid structures. **h** Prognostic value of tertiary lymphoid structures (TLS) in cervical cancer as indicated by Kaplan–Meier curves of progression-free survival (PFS). *P* value was based on log-rank test.

We explored the prognostic impact of baseline immune markers in cervical cancer and found CD8+ T cell infiltration as the most promising candidate to be associated with beneficial clinical outcome regardless of neoadjuvant treatment (Supplementary Fig. 3a). We further considered the combination of immunologic features and performed unsupervised hierarchical clustering of eight attributes. Four subgroups were revealed and arbitrarily designated as cluster 1 (16.8%), cluster 2 (37.9%), cluster 3 (26.3%), and cluster 4 (18.9%) (Fig. 2a). Cluster 1 (termed ‘immuno-active’) exhibited marked positivity for nearly all IHC markers other than CD56 and PD-1, hence resembling typical immunoreactive tumours (Fig. 2b). Cluster 2 (termed ‘immuno-medial’) showed moderate levels of immune contents (Fig. 2c). Cluster 3 (termed ‘immuno-deficient’) represented the immunologically inert prototype with low immune cell densities (Fig. 2d). Cluster 4 (termed ‘immuno-NK’) was uniquely defined by prominent CD56+ NK cells (Fig. 2e). We found that the ‘immuno-deficient’ group (cluster 3) had relatively shorter PFS than the ‘immuno-active’ group (cluster 1), and the ‘immuno-medial’ group (cluster 2) displayed intermediate risk of relapse (Fig. 2f). Interestingly, patients categorised as ‘immuno-NK’ (cluster 4) demonstrated a PFS advantage compared to those in the ‘immuno-deficient’ and ‘immuno-medial’ classes (Fig. 2f). Of note, we discovered tertiary lymphoid structures (TLSs) characterised by ectopic intratumoural aggregates of B and T lymphocytes (Fig. 2g and Supplementary Fig. 1b), which preferentially existed in the ‘immuno-active’ tumours (45.8%) and tended to correlate with improved PFS (Fig. 2h). Therefore, the magnitude and composition of baseline immune infiltrates aided the stratification of cervical cancer patients into distinct molecular subtypes with prognostic relevance.

### Immune augmentation upon NACT

**Fig. 3:**
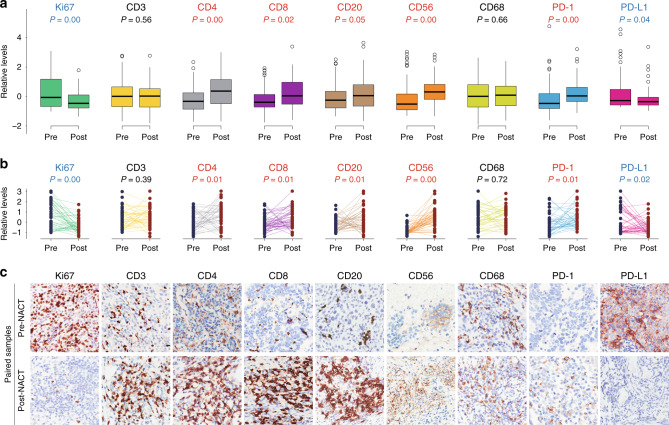
Immune augmentation upon neoadjuvant chemotherapy. **a** Relative levels of immunohistochemical staining intensities in all pre-NACT (*n* = 92) and post-NACT (*n* = 60) cervical tumour samples. Blue labels: significant decrease; red labels: significant increase; black labels: statistically unchanged. **b** Relative levels of immunohistochemical staining intensities in paired pre-NACT (*n* = 43) and post-NACT (*n* = 43) cervical tumour samples. Blue labels: significant decrease; red labels: significant increase; black labels: statistically unchanged. **c** Representative images (×20) of immunohistochemical staining in paired pre- and post-NACT cervical tumour samples.

Sixty-nine patients with locally advanced disease were first dosed with primary chemotherapeutic regimens and subsequently evaluated to further receive surgical resection (62 patients) or chemoradiotherapy (7 patients). This neoadjuvant setting, although controversial,^6^ offered an unprecedented opportunity to investigate the potential impact of conventional systemic intervention on tumour microenvironment for rational combination with immunotherapeutics and to explore the predictive determinants of chemosensitivity for patient-tailored medicine. To this end, we collected 60 specimens from debulking surgery after platinum-based doublets (mostly cisplatin) and carried out IHC assessment using the same aforementioned antibody panel. Compared to the baseline (92 samples), cervical cancer following chemotherapy (60 samples) experienced a significant reduction in Ki67 and PD-L1 positivity (Fig. 3a), in line with drug-invoked tumour cell death. By contrast, the densities of multiple immune markers, including CD4, CD8, CD20, CD56, and PD-1, were evidently increased in chemo-treated samples (Fig. 3a). Treatment-conferred enrichment of CD4+, CD8+, CD20+, and CD56+ TIL was verified by performing paired analysis (Fig. 3b) and inspecting representative IHC images (Fig. 3c) in the 43 cases with matched pre- and post-NACT sections. Although immunomodulatory effects of NACT were considerably variable among these 43 individuals, an expansion of each immune cell population was noted in >50% of the patients without exception (Supplementary Fig. 4a). Of particular relevance, we also observed CD14+ myeloid cell depletion by NACT (Supplementary Fig. 4b), which was shown to foster robust T cell reactivity in HPV16-based vaccination.^53,54^ In addition, TLSs were markedly induced and arose de novo in some cases (Supplementary Fig. 4c). These data suggested that NACT fostered pronounced immune augmentation in cervical cancer.

### Patterns of immune augmentation upon NACT

**Fig. 4:**
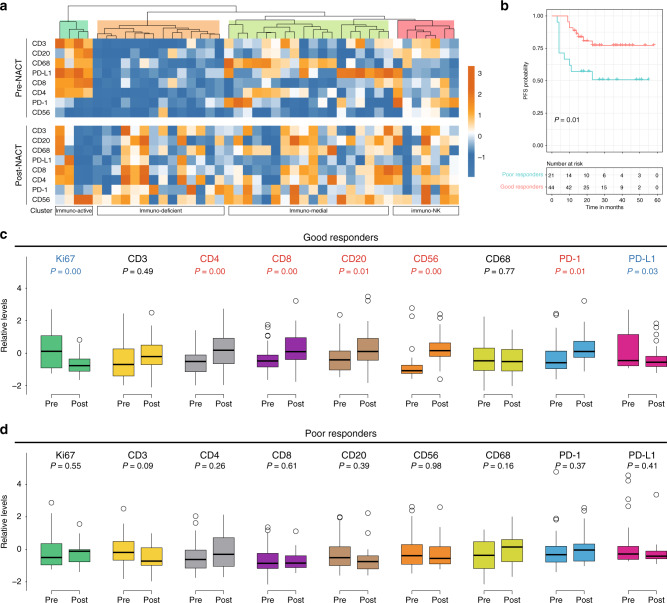
Patterns of immune augmentation upon neoadjuvant chemotherapy. **a** Heatmap for the comparison of immune infiltration in paired pre-NACT (*n* = 43) and post-NACT (*n* = 43) samples clustered by immune subtypes. **b** Kaplan–Meier curves of progression-free survival (PFS) for good and poor responders to NACT. All patients who received neoadjuvant chemotherapy and had clinical follow-up information were included in the analysis. *P* value was based on log-rank test. **c** Relative levels of immunohistochemical staining intensities in pre- and post-NACT tumour samples of 44 good responders. Blue labels: significant decrease; red labels: significant increase; black labels: statistically unchanged. **d** Relative levels of immunohistochemical staining intensities in pre- and post-NACT tumour samples of 21 poor responders. Blue labels: significant decrease; red labels: significant increase; black labels: statistically unchanged.

We sought to delineate the overall patterns of immunostimulation by NACT in more detail. Analogous to the earlier immune profiles, the NACT cohort with paired samples (43 patients) could be hierarchically divided into ‘immuno-active’, ‘immuno-medial’, ‘immuno-deficient’, and ‘immuno-NK’ subtypes as well. The most striking TIL accumulation occurred in the initially classified ‘immuno-deficient’ tumours (Fig. 4a). Chemotherapeutic-elicited immunogenic phenotype was also manifested by frequent gain of CD56+ NK cells across all four molecular clusters (Fig. 4a). We further compared TIL levels with respect to the clinical outcome by segregating NACT-treated patients (65 out of 69 evaluable) into good responders (with CR or PR) and poor responders (with SD or PD) (Supplementary Fig. 5). As expected, 44 good responders exhibited better PFS than 21 poor responders (Fig. 4b). Of interest, decreased Ki67 and PD-L1 signals, as well as intensified CD4, CD8, CD20, CD56, and PD-1 staining, were specifically observed in good responders (Fig. 4c) but not in poor responders (Fig. 4d). Although the limited number of cases and events did not allow for definitive assessment on the predictive value of immune augmentation, elevated abundance of diverse lymphatic cell populations, similar to the TIL-enriched status regardless of medication, tended to be positively associated with chemotherapy response (Supplementary Fig. 6). Collectively, the immunomodulatory action of NACT was affected by both baseline immunity and therapeutic efficacy.

### Evaluation of immunologic properties with RNA-seq

**Fig. 5:**
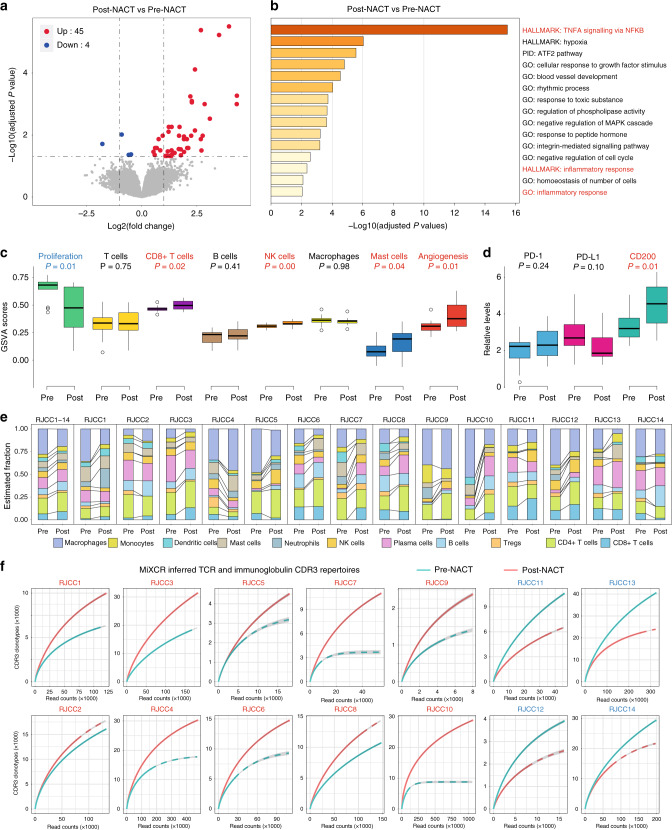
Evaluation of immunologic properties with RNA sequencing. **a** Volcano plot of differential gene expression in paired pre-NACT (*n* = 14) and post-NACT (*n* = 14) cervical tumour samples as assessed by RNA-seq. There were 45 upregulated genes and 4 downregulated genes in post-NACT samples relative to pre-NACT samples (adjusted *P* value <0.05). **b** Gene ontology and pathway analyses of differentially expressed genes using Metascape. **c** Comparison of GSVA scores for the indicated gene signatures in paired pre-NACT (*n* = 14) and post-NACT (*n* = 14) cervical tumour samples. Blue labels: significant decrease; red labels: significant increase; black labels: statistically unchanged. **d** Relative gene expression levels of PD-1, PD-L1, and CD200 as measured by RNA-seq in paired pre-NACT (*n* = 14) and post-NACT (*n* = 14) cervical tumour samples. **e** The relative abundance of diverse immune cell infiltrates in paired pre-NACT (*n* = 14) and post-NACT (*n* = 14) cervical tumour samples was estimated by CIBERSORT analysis using the RNA-seq data. **f** MiXCR inferred TCR and immunoglobulin CDR3 repertoires in paired pre-NACT (*n* = 14) and post-NACT (*n* = 14) tumour samples of each cervical cancer patient. Blue labels: the number of CDR3 clonotypes was decreased in post-NACT tumour relative to pre-NACT tumour; red labels: the number of CDR3 clonotypes was increased in post-NACT tumour relative to pre-NACT tumour.

We leveraged the RNA-seq data of 14 fresh-frozen sample pairs (RJCC1–14) to validate the relationship between antitumour immunity and neoadjuvant treatment. NACT caused discrepant changes of gene expression in each patient and, across the cohort, resulted in 45 upregulated and 4 downregulated transcripts (Fig. 5a and Supplementary Fig. 7). Gene ontology and pathway analyses of differentially expressed genes pinpointed multiple significantly altered modules related to immune activation upon chemotherapy, i.e. ‘TNFA signalling via NFKB’ and ‘inflammatory response’ (Fig. 5b). We conducted gene set variation analysis (GSVA) with predefined transcriptional signatures for a range of biological processes and found that cell proliferation was indeed impaired, whereas relative amounts of CD8+ T cells, NK cells, and mast cells were increased following NACT (Fig. 5c). In addition, by specifically analysing various immune checkpoints (Supplementary Fig. 8), we identified a significant upregulation of the immunosuppressive CD200 molecule (Fig. 5d), which might serve as a potential immunotherapeutic target in chemo-treated cervical cancer. CIBERSORT algorithm confirmed the enlarged fractions of CD4+ and CD8+ T cell subsets in the majority of residual lesions (Fig. 5e); on the contrary, there were relatively fewer remaining macrophages and T regulatory cells, both considered negative mediators of immune function. Finally, the deep transcriptome sequencing enabled computational inference of antigen receptor diversities reflected by T cell receptor and immunoglobulin repertoires using the MiXCR pipeline.^51^ More complementarity determining region 3 clonotypes were extracted from chemo-exposed tumours in 10 out of 14 sequenced subjects (Fig. 5f), implying enhanced lymphocyte infiltration. We concluded that the RNA-seq experiment substantially verified our IHC findings of chemotherapy-coupled immunostimulation in cervical cancer.

## Discussion

In this study, by integrating IHC and RNA-seq analysis, we presented a rational approach for detailed interrogation of immune microenvironment in a large series of cervical cancer. Our data revealed divergent baseline immunologic states that stratified patients into distinctive prognostic subgroups. Brief exposure and clinical response to NACT seemingly incited a favourable reshaping of antitumour immunity against cervical carcinoma. These findings not only hold promise to better understand the impact of tumour–immune interactions on disease behaviour and management but also provide the foundation to investigate synergistic treatment options of combining conventional chemotherapy with immunotherapeutic agents.

We employed a robust in silico deconvolution framework to estimate the immune constituents from bulk gene expression profiles,^50^ which indicated the highest degree of infiltrating B cells, T cells, NK cells, and macrophages in cervical tumours. It is noteworthy that the computational measurements were at best approximate, and a definitive cellular composition and abundance can be conceivably resolved using single-cell RNA-seq technology in the future.^55,56^ Nevertheless, these prevalent TIL populations were independently validated by immunostainings and collectively segregated samples into four molecular subtypes. As with numerous other cancer types,^57^ cervical malignancies were vastly heterogeneous in the breadth of immune cell infiltration. Remarkably, we found that B cells and T cells sporadically formed into TLSs, which were reported to play a direct role in the priming of antitumour immunity.^58–62^ In addition, a unique subset of patients was revealed to contain disproportionate intratumoural NK cells and has exceptionally inferior risk of disease progression compared to other molecular subtypes. This observation accords with the notion that NK cells are key to cancer immunosurveillance as both cytolytic effectors of the innate immune system and emerging regulators of the adaptive immune cascade.^63,64^ Taken together, our in-depth characterisation of the immune portraits reinforced the immunogenic nature of virally driven cervical cancer.

Chemotherapy, including the mainstay cisplatin in cervical cancer, has traditionally been considered largely immunosuppressive due to its direct haematologic toxicity. However, such view is challenged by cumulative evidence showing that it can enhance certain facets of locoregional immune response in a variety of human cancers.^21–23,29,65^ Along this line, we discovered that preoperative chemotherapy indeed converted cervical lesion into a site permissive for antitumour immunity, as exemplified by selective enrichment of CD4+, CD8+, CD20+, and CD56+ TIL. The molecular mechanisms underlying the inflammatory effects of cytotoxic chemotherapeutics have been predominantly attributed to the drug-evoked immunogenic cell death, involving for instance surface calreticulin exposure,^66^ HMGB1 secretion,^67^ autophagic ATP release,^68^ NLRP3 inflammasome activation,^69^ cytokine production,^70^ and instigation of antigen-presenting dendritic cells.^71^ Alternatively, recent work showed that standard chemotherapy was able to reduce immunosuppressive myeloid cells and enhance T cell responses to therapeutic HPV16 vaccine in cervical cancer.^53,54^ Of note, myriad preclinical and clinical studies have also unveiled differential immunostimulatory capacities of different chemotherapeutic agents.^72,73^ Given the pleiotropic functions of chemotherapy, additional work is deserved to fully elucidate the mechanistic determinants responsible for the augmenting immune activities.

Although ongoing trials with immune checkpoint inhibitors in cervical cancer have shown early promising outcome, clinical responses are generally modest and variable.^74^ A disappointing 3% objective response rate (ORR) was observed in a Phase 1/2 trial of 42 women who received ipilimumab (anti-CTLA-4) as monotherapy.^75^ In KEYNOTE-028 with pembrolizumab (anti-PD-1), ORR was 17% and median duration of response was merely 5.4 months.^76^ Most recently, the KEYNOTE-158 Phase 2 basket trial presented an interim ORR of 12.2%, leading to the accelerated approval of pembrolizumab in advanced PD-L1-positive cervical cancer.^77^ Overall, the potency of immune-based regimens is limited in unselected patient populations and should be tailored according to clinicopathological or molecular attributes. Illuminated by current study, we propose the following paradigm shift toward precision immunotherapy for cervical cancer. The ‘immuno-active’ tumours may experience spontaneous immunogenicity considering pronounced basal lymphocyte infiltration and PD-L1 expression and are likely poised to benefit from immunomodulatory medicine regardless of chemotherapeutic education. For the ‘immuno-medial’ or ‘immuno-active’ subtypes, combined or induction chemotherapy is a plausible option to provoke iatrogenic immunogenicity. The ‘immuno-NK’ cluster illustrates an opportunity for pharmacological inhibition of NK cell checkpoints.^78^ Therefore, an improved understanding of the immune status at baseline and upon specific treatments could yield valuable insights into more optimised and efficacious therapeutic modalities of cervical cancer.

Several limitations of this exploratory study have to be acknowledged. First, our investigations were retrospective in nature with potential biases owing to missing clinical records and unpredictable tissue availability. Second, these preliminary results stemmed from one patient cohort in a single institution without internal and external validation data sets. Third, we mainly relied on conventional protein markers to define immune cell types and ideally the corroborative RNA-seq analyses should have included a larger number of subjects. Finally, the prognostic and predictive significance of chemo-induced TIL remodelling was underpowered to determine and future efforts with adequate sample size are warranted in this respect.

## Conclusions

In summary, we provided for the first time a comprehensive snapshot of baseline immunologic features within cervical tumour microenvironment and further uncovered the association between NACT effects and an immunostimulatory phenotype. Expanded studies in the prospective setting are required to verify these findings, which may have clinical implications for tailoring immune-based treatment in women with cervical cancer.

## Supplementary information

Supplementary files

## Data Availability

All reagents used in this study were commercially available. The RNA sequencing data have been deposited in NCBI BioProject database (http://www.ncbi.nlm.nih.gov/bioproject/) under the accession number SRP173984.
