# Supplementary material for: Baseline immunity and impact of chemotherapy on immune microenvironment in cervical cancer
Source: Br J Cancer. 2020 Oct 22;124(2):414–24. doi: 10.1038/s41416-020-01123-w (PMC7852680; doi:10.1038/s41416-020-01123-w)
Supplement: Supplementary file 1 — Supplementary files [file 41416_2020_1123_MOESM1_ESM.pdf]

Supplementary Figure 1.

A

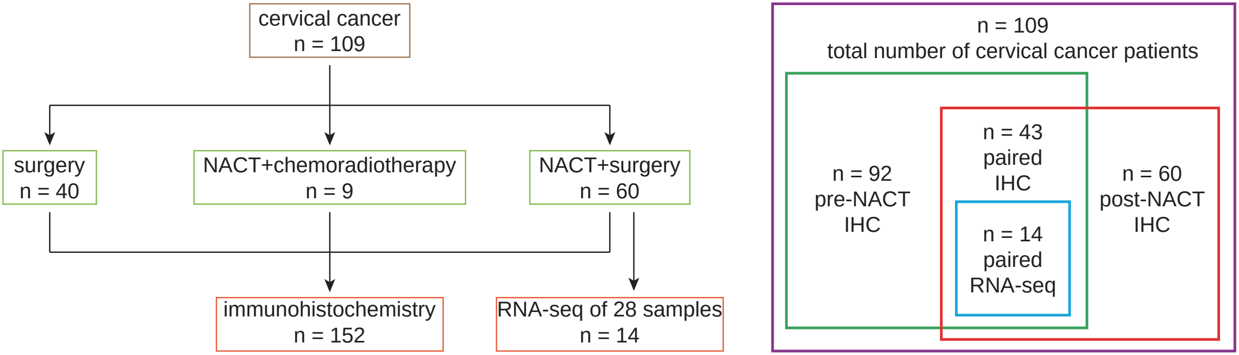

B

Input Sample	B cells naive	B cells memory	Plasma cells	T cells CD8	T cells CD4 naive	T cells CD4 memory resting	T cells CD4 memory activated	T cells follicular helper	T cells regulatory (Tregs)	T cells gamma delta	NK cells resting	NK cells activated	Monocytes	Macrophages M0	Macrophages M1	Macrophages M2	Dendritic cells resting	Dendritic cells activated	Mast cells resting	Mast cells activated	Eosinophils	Neutrophils
RJCC1-N	0	0.043	0.129	0.011	0.057	0.056	0	0	0.027	0	0	0.041	0.01	0.024	0.036	0.298	0	0.049	0	0.165	0	0.054
RJCC1-C	0	0.023	0.112	0.034	0.037	0.072	0	0	0.035	0	0	0.037	0.047	0.065	0.016	0.09	0.01	0.069	0	0.14	0	0.213
RJCC2-N	0	0.112	0.223	0.02	0.02	0.237	0	0	0.044	0	0.043	0.041	0.038	0.012	0.018	0.04	0.031	0.02	0.102	0	0	0
RJCC2-C	0	0.169	0.193	0.042	0.099	0.115	0.004	0	0	0	0.075	0	0.017	0.048	0.011	0.076	0	0.062	0.079	0.004	0	0.007
RJCC3-N	0	0.073	0.29	0.058	0.073	0.17	0	0	0.044	0	0.049	0.024	0.043	0.016	0.064	0.021	0	0.001	0.061	0	0	0.012
RJCC3-C	0	0.125	0.201	0.133	0	0.293	0	0	0.015	0	0.021	0.068	0.054	0	0.018	0.018	0	0.001	0.02	0.034	0	0
RJCC4-N	0	0.018	0.078	0.059	0.028	0.05	0	0	0.112	0	0.039	0.063	0	0.21	0.011	0.123	0	0.025	0	0.126	0	0.059
RJCC4-C	0.004	0.015	0.075	0.035	0.005	0.047	0.001	0	0.028	0	0.033	0	0.035	0.386	0.023	0.048	0	0.02	0	0.173	0	0.073
RJCC5-N	0	0.014	0.021	0.069	0	0.202	0.04	0	0	0	0.029	0.053	0.018	0	0.08	0.399	0.005	0.005	0.054	0	0	0.009
RJCC5-C	0	0.047	0.017	0.02	0	0.267	0.044	0	0.001	0	0.138	0.019	0.041	0.065	0.088	0.132	0.002	0.031	0.015	0	0.013	0.06
RJCC6-N	0	0.155	0.109	0.141	0.024	0.169	0	0	0.015	0	0.075	0.023	0.028	0	0.032	0.128	0	0.054	0.016	0	0	0.031
RJCC6-C	0	0.168	0.058	0.145	0	0.273	0.024	0	0.02	0	0.045	0.017	0.035	0	0.013	0.055	0	0.002	0	0.126	0	0.019
RJCC7-N	0	0.041	0.008	0	0.088	0.148	0	0	0.062	0	0.028	0.016	0.052	0.128	0.003	0.071	0	0.113	0	0.155	0	0.087
RJCC7-C	0	0.098	0.131	0.131	0	0.203	0	0	0.063	0	0.004	0.082	0.044	0	0.011	0.103	0	0	0.128	0	0	0
RJCC8-N	0	0.177	0.101	0.108	0.039	0.114	0	0	0.05	0	0.072	0	0	0.027	0.052	0.081	0	0.09	0	0.016	0	0.072
RJCC8-C	0	0.125	0.202	0.093	0	0.276	0	0	0.017	0	0.034	0.002	0.055	0.001	0.005	0.083	0	0.012	0.059	0.011	0	0.027
RJCC9-N	0	0.022	0.008	0.001	0.019	0.149	0	0	0	0	0.012	0.022	0.196	0.317	0.008	0.072	0	0	0	0.052	0	0.123
RJCC9-C	0	0.036	0.02	0.007	0	0.283	0	0	0	0	0.049	0.029	0.106	0.153	0.013	0.272	0	0	0.016	0.016	0	0
RJCC10-N	0	0.04	0.082	0	0.031	0.087	0	0	0	0	0.023	0	0	0.412	0	0.12	0	0.08	0	0.116	0	0.01
RJCC10-C	0	0.151	0.217	0.076	0.014	0.192	0	0	0.05	0	0.031	0.007	0.03	0.076	0.015	0.082	0	0.002	0	0.056	0	0
RJCC11-N	0	0.108	0.165	0.15	0	0.215	0.001	0	0.048	0	0.048	0	0.067	0	0.035	0.149	0.001	0.01	0	0	0	0.003
RJCC11-C	0	0.096	0.112	0.233	0	0.157	0	0.005	0.042	0	0	0.127	0.075	0	0.045	0.098	0.006	0.003	0.002	0	0	0
RJCC12-N	0	0	0.033	0.102	0	0.082	0.072	0	0.04	0	0.106	0	0.03	0.027	0.166	0.205	0	0.023	0	0.052	0	0.063
RJCC12-C	0	0.05	0.054	0.168	0	0.159	0.091	0	0	0	0.14	0	0.055	0	0.164	0.086	0	0.003	0.03	0	0	0
RJCC13-N	0	0.092	0.206	0.122	0	0.122	0	0	0.048	0	0.03	0.045	0.032	0.055	0.055	0.112	0	0.007	0.053	0	0	0.022
RJCC13-C	0	0.041	0.289	0.114	0	0.178	0	0	0.02	0	0.025	0.003	0.093	0.006	0	0.046	0	0.027	0.144	0.012	0	0
RJCC14-N	0	0.09	0.216	0.069	0.024	0.108	0	0	0.037	0	0.035	0.031	0.002	0.095	0.036	0.174	0	0.062	0.009	0	0	0.011
RJCC14-C	0	0.226	0.2	0.017	0	0.131	0	0.001	0	0	0.01	0.037	0.023	0	0.052	0.224	0	0.068	0.007	0	0	0.005

Supplementary Figure 1.
A. Schematic overview of the study and the number of patients analyzed by IHC or RNA-seq.
B. The relative abundance of diverse immune cell infiltrates in pre- (N) and post-NACT (C) cervical tumor samples was estimated by CIBERSORT analysis using the RNA-seq data. N: naïve; C: chemotherapy.

Supplementary Figure 2.

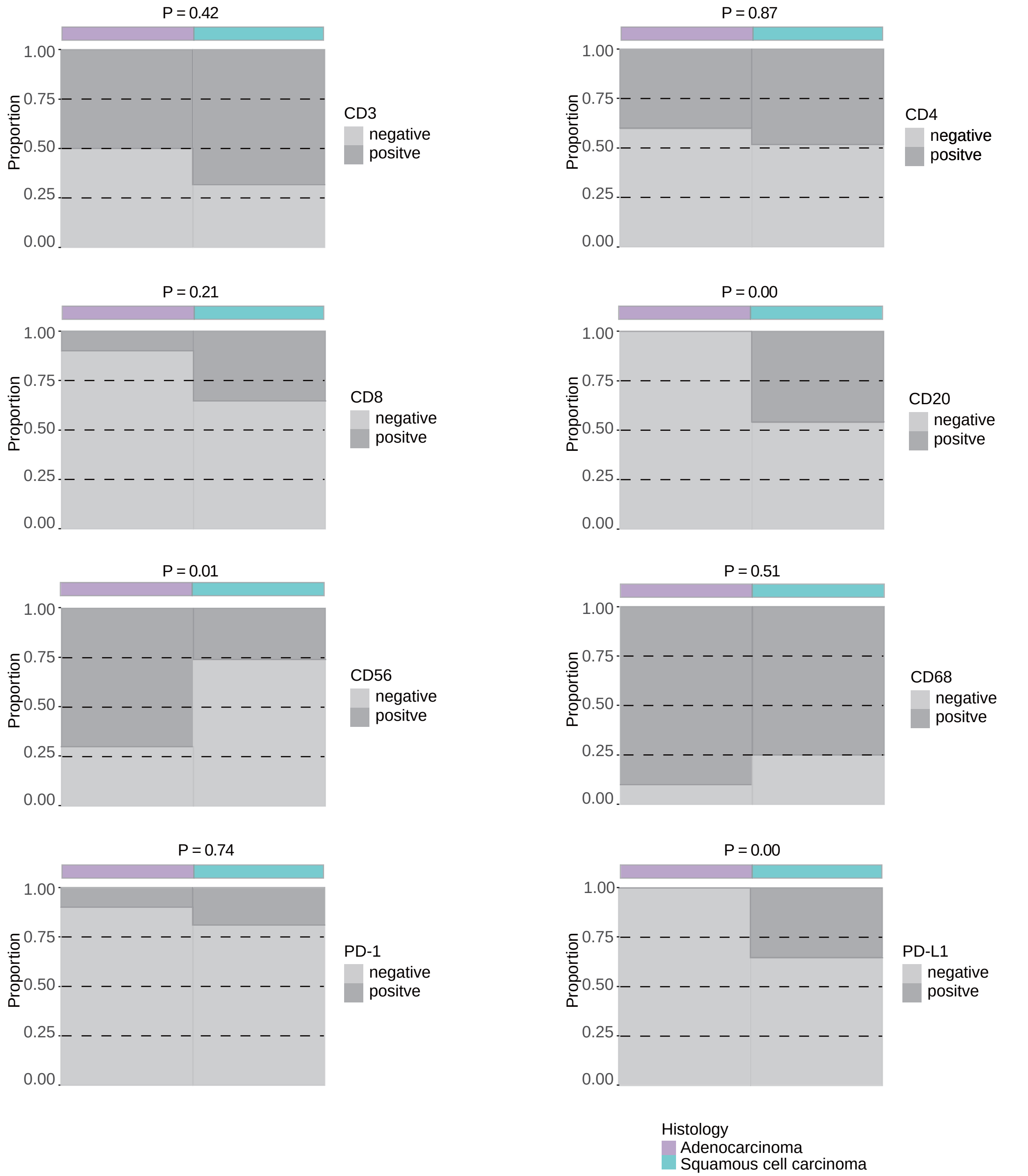

Supplementary Figure 2.
The proportion of cervical tumors showing positive or negative staining of indicated immune markers. The samples were segregated on the basis of different histology. Specifically, the studied cohort included 10 cervical adenocarcinomas and 81 squamous cell carcinomas.

Supplementary Figure 3.

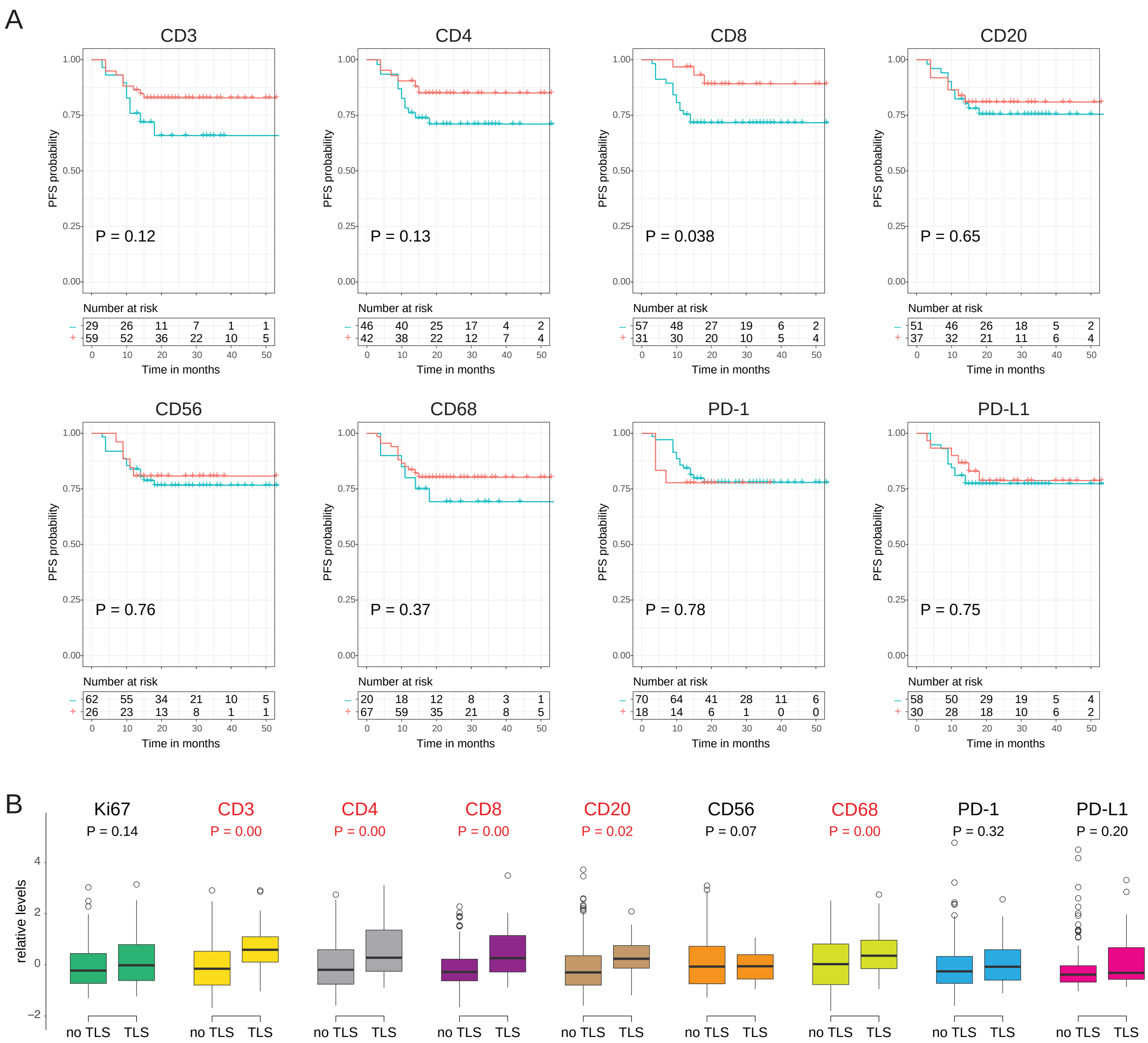

Supplementary Figure 3.
A. Prognostic value of each immune marker in cervical cancer as indicated by Kaplan-Meier progression-free survival (PFS) analysis. We used semi-quantitative IHC scores to define the positive (‘+’, ‘++’, ‘+++’) or negative (‘-’) group. P-value was based on the log-rank test.
B. Relative levels of immunohistochemical staining intensities in cervical tumor samples with or without tertiary lymphoid structures (TLS). Red labels: significant increase; black labels: statistically unchanged.

Supplementary Figure 4.

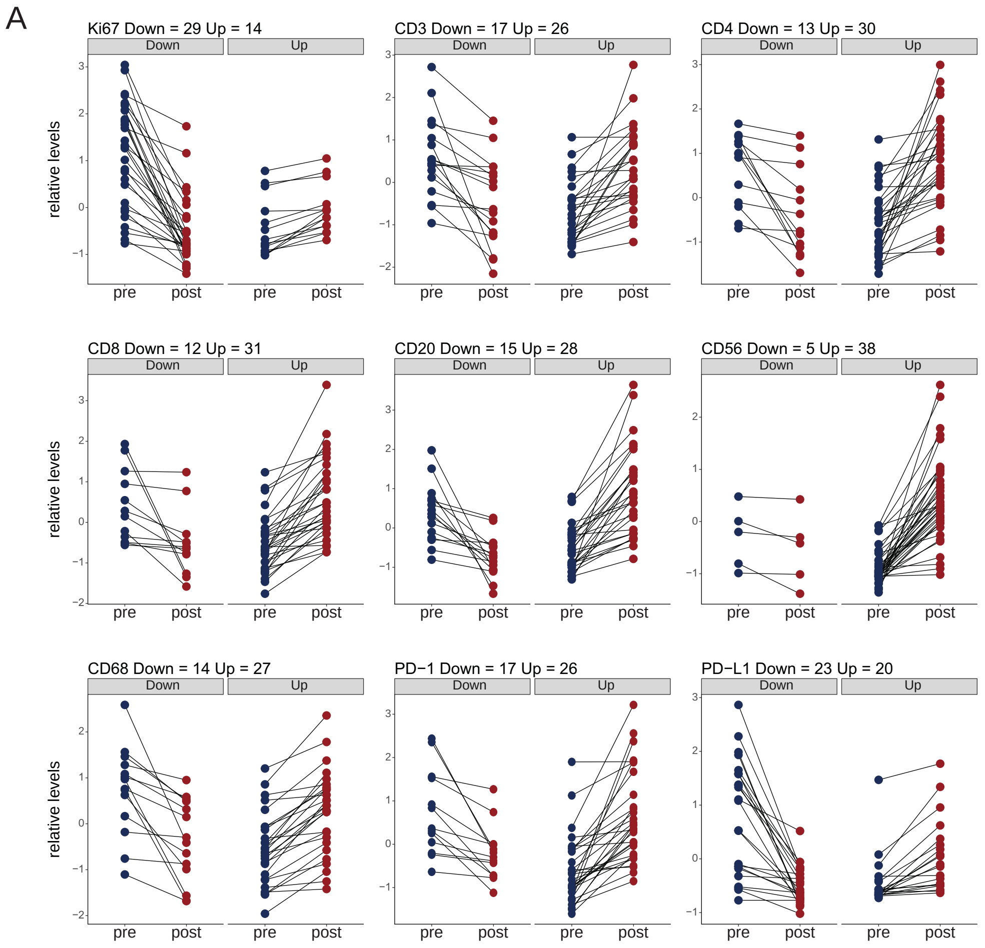

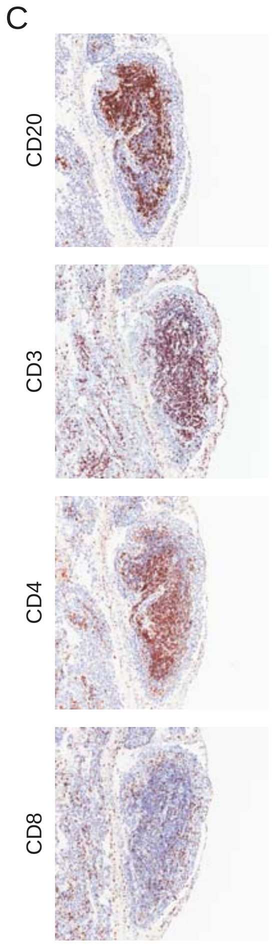

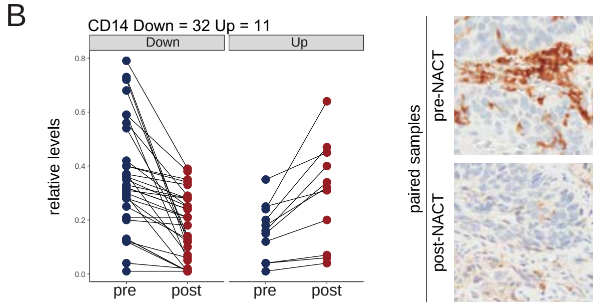

Supplementary Figure 4.
A. Relative levels of immunohistochemical staining intensities in paired pre- and post-NACT cervical tumor samples. A total of 43 patients were grouped separately according to different trends of intensity changes in post-NACT tumor relative to pre-NACT tumor.
B. Relative levels of CD14+ myeloid cells in paired pre- and post-NACT cervical tumor samples.
C. Immunohistochemical staining of the indicated immune markers illustrated the de novo occurrence of tertiary lymphoid structures in post-NACT tumors.

Supplementary Figure 5.

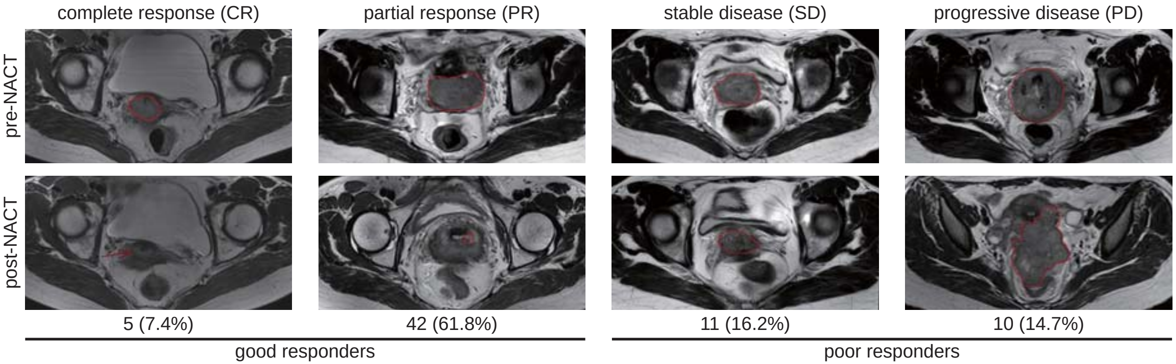

Supplementary Figure 5.
NACT response was assessed by magnetic resonance imaging (MRI). Representative images and patient numbers for CR, PR, SD and PD were shown. Patients with CR or PR were defined as good responders, and patients with SD or PD were defined as poor responders.

Supplementary Figure 6.

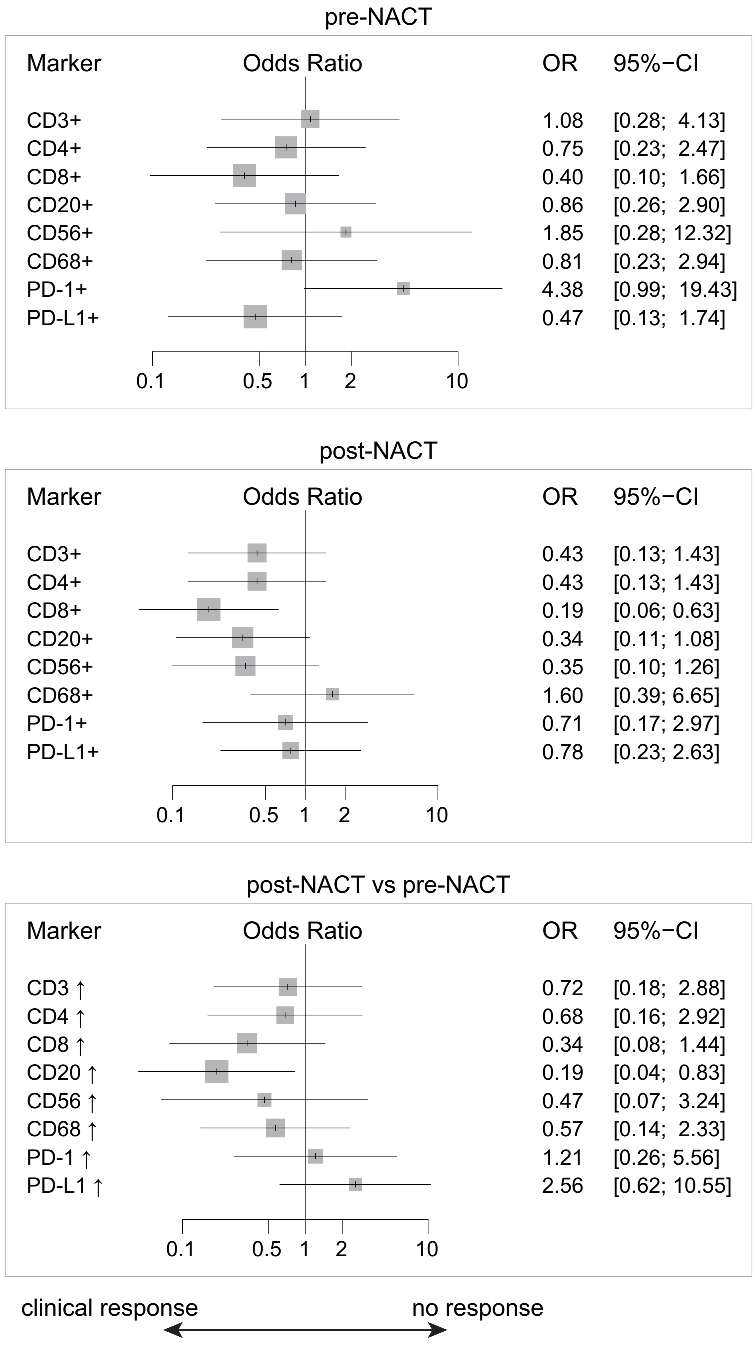

Supplementary Figure 6.
The relative contribution of evaluated immune markers to chemotherapy response was indicated by the odds ratio with 95% confidence interval (CI) shown in the forest plot. All 69 patients who received neoadjuvant chemotherapy were included in the pre-NACT group, and all 60 patients with tissue available after neoadjuvant chemotherapy were included in the post-NACT group. For post-NACT vs pre-NACT comparison, the 43 cases with matched samples were used.

Supplementary Figure 7.

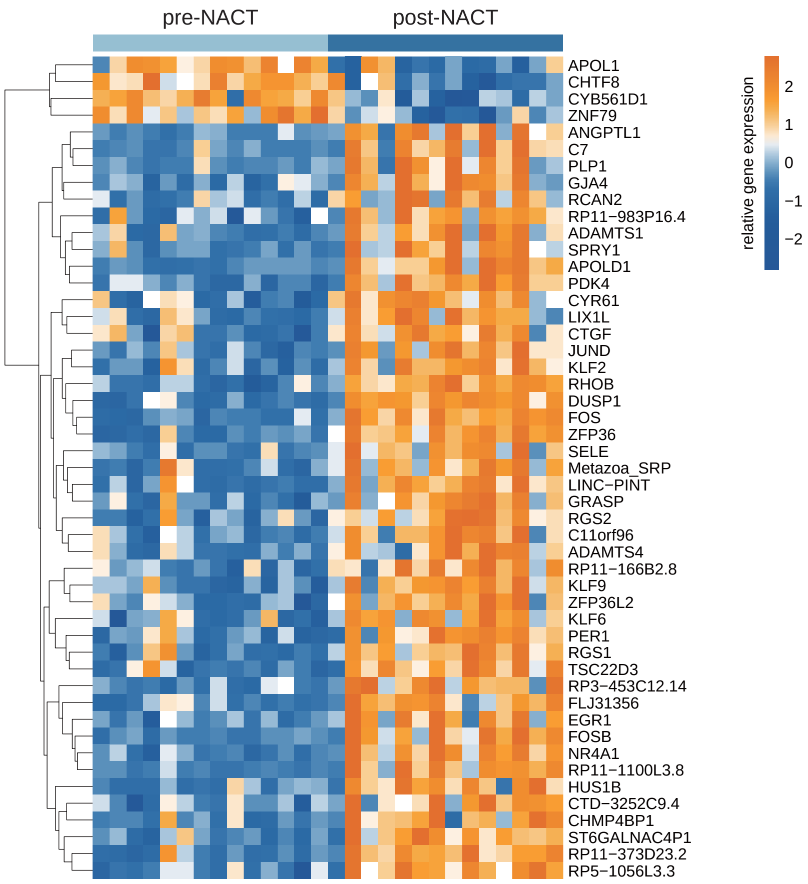

Supplementary Figure 7.
Heatmap of differential gene expression in paired pre- and post-NACT cervical tumor samples as assessed by RNA-seq.

Supplementary Figure 8.

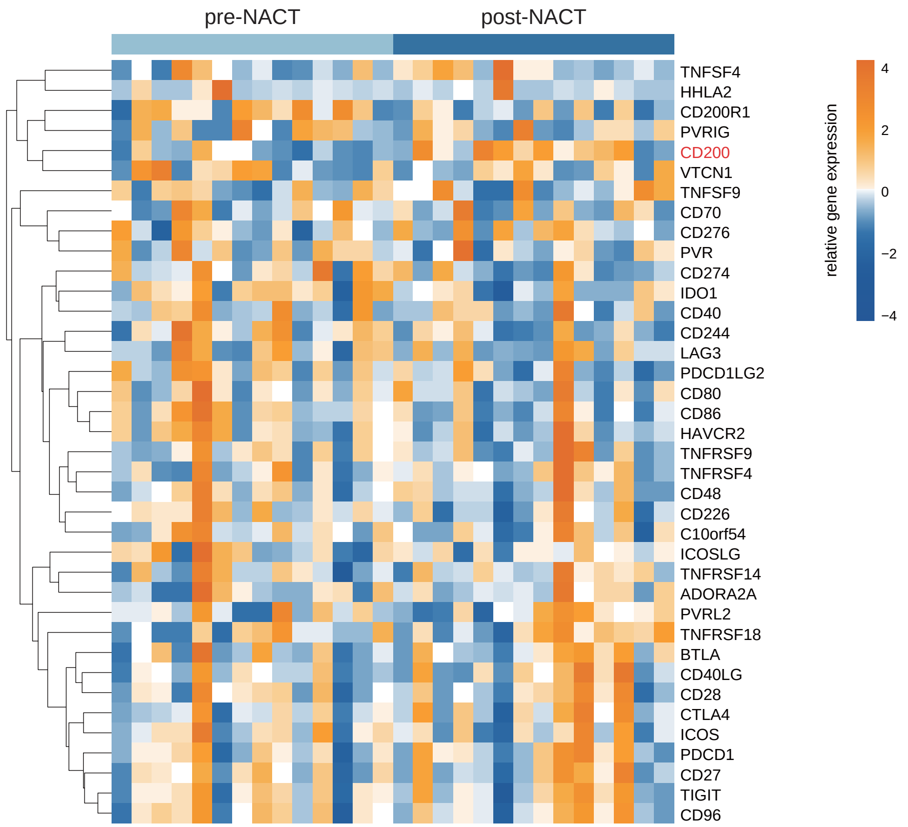

Supplementary Figure 8.
Heatmap of gene expression for immune checkpoints in paired pre- and post-NACT cervical tumor samples as assessed by RNA-seq.

Supplementary Table 1. Clinico-pathological characteristics of patients

Characteristic	N (%)
No. of patients	109
Median age (years)	52
Average serum SCC at entry (kU/L)	20.6
**FIGO stage**	
IA1	1 (0.9)
IB1	31 (28.4)
IB2	18 (16.5)
IIA1	12 (11.0)
IIA2	35 (32.1)
IIB	11 (10.1)
IVA	1 (0.9)
**Histopathology**	
squamous cell carcinoma	98 (89.9)
adenocarcinoma	10 (9.2)
neuroendocrine carcinoma	1 (0.9)
**Management**	
radical hysterectomy	40 (36.7)
NACT + surgery/chemoradiotherapy	69 (63.3)
**Chemotherapy regimens**	
platinum + taxanes	66 (95.7)
platinum + etoposide	1 (1.4)
platinum + irinotecan	1 (1.4)
platinum + 5-FU	1 (1.4)
**Chemotherapy outcome**	
complete remission (CR)	5 (7.4)
partial remission (PR)	42 (61.8)
stable disease (SD)	11 (16.2)
progressive disease (PD)	10 (14.7)
**Methodological approaches**	
immunohistochemistry	109 (100)
RNA sequencing	14 (12.8)

Supplementary Table 2. Antibodies used for the study

Antibody	Target	Source	Clonality	Catalog#	Manufacturer	No. of analyzed specimens
Anti-CD3	pan T cells	Rabbit	monoclonal	Kit-0003	MXB biotechnologies	152
Anti-CD4	CD4+ helper T cells	Rabbit	monoclonal	RMA-0620	MXB biotechnologies	152
Anti-CD8	CD8+ cytotoxic T cells	Rabbit	monoclonal	RMA-0514	MXB biotechnologies	152
Anti-CD20	B cells	Mouse	monoclonal	Kit-0001	MXB biotechnologies	152
Anti-CD56	natural killer cells	Mouse	monoclonal	Kit-0028	MXB biotechnologies	152
Anti-CD68	macrophages	Mouse	monoclonal	Kit-0026	MXB biotechnologies	151
Anti-PD-1	programmed death-1	Mouse	monoclonal	MAB-0734	MXB biotechnologies	152
Anti-PD-L1	programmed death-ligand 1	Mouse	monoclonal	RMA-0732	MXB biotechnologies	152
Anti-Ki-67	proliferating cells	Mouse	monoclonal	Kit-0005	MXB biotechnologies	152
Anti-CD14	myeloid cells	Rabbit	monoclonal	75181S	CST	86
